# The School Anxiety Scale-Teacher Report (SAS-TR): translation and psychometric properties of the Iranian version

**DOI:** 10.1186/1471-244X-12-82

**Published:** 2012-07-18

**Authors:** Zahra Hajiamini, Ashraf Mohamadi, Abbas Ebadi, Ali Fathi- Ashtiani, Mahmoud Tavousi, Ali Montazeri

**Affiliations:** 1 Behavioral Sciences Research Center and School of Nursing, Baqiyatallah University of Medical Sciences, Tehran, Iran; 2 School of Nursing, Bagiyatallah University of Medical Sciences, Tehran, Iran; 3 Behavioral Sciences Research Center, Bagiyatallah University of Medical Sciences, Tehran, Iran; 4 Health Education and Promotion Research Group, Health Metrics Research Center, Iranian Institute for Health Sciences Research, ACECR, Tehran, Iran; 5 Mental Health Research Group, Health Metrics Research Center, Iranian Institute for Health Sciences Research, ACECR, Tehran, Iran

## Abstract

**Background:**

The School Anxiety Scale-Teacher Report (SAS-TR) was designed to assess anxiety in children at school. The SAS-TR is a proxy rated measure and could assess social anxiety, generalized anxiety and also gives a total anxiety score. This study aimed to translate and validate the SAS-TR in Iran.

**Methods:**

The translation and cultural adaptation of the original questionnaire were carried out in accordance with the published guidelines. A sample of students participated in the study. Reliability was estimated using internal consistency and test-retest analysis. Validity was assessed using content validity. The factor structure of the questionnaire was extracted by performing both exploratory and confirmatory factor analyses.

**Results:**

In all 200 elementary students aged 6 to 10 years were studied. Considering the recommended cut-off values, overall the prevalence of high anxiety condition in elementary students was found to be 21 %. Cronbach's alpha coefficient for the Iranian SAS-TR was 0.92 and intraclass correlation coefficient (ICC) was found to be 0.81. The principal component analysis indicated a two-factor structure for the questionnaire (generalized and social anxiety) that jointly accounted for 55.3 % of variances observed. The confirmatory factory analysis also indicated a good fit to the data for the two-latent structure of the questionnaire.

**Conclusion:**

In general the findings suggest that the Iranian version of SAS-TR has satisfactory reliability, and validity for measuring anxiety in 6 to 10 years old children in Iran. It is simple and easy to use and now can be applied in future studies.

## Background

Anxiety disorders represent one of the most prevalent childhood psychiatric disorders [1,2]. It has been estimated that about 5 to 20 % of children and adolescents suffer from anxiety disorder. A high rate for co-morbid depression and anxiety ranging from 15.9 to 61.9 % has also been documented [3-5]. Recent epidemiological studies have suggested that between 8 to 12 % of children suffer from some type of anxiety disorder that is sufficiently severe to interfere with their daily functioning [6]. Anxiety disorders have the earliest median age of onset of all psychiatric disorders [7,8]. Unfortunately, despite prevalence and severity of anxiety disorders, only approximately 30 % of children with these disorders receive treatment [9,10]. Although a mild to moderate level of anxiety is crucial for learning and can promote better adjustment, excessive anxiety can be detrimental to children’s physical and psychological health [9-11]. If left untreated, anxiety disorders in youth may lead to chronic emotional problems and substance abuse, and anxious symptoms often worsen over time [5,12].

Two demographic factors are important when investigating anxiety in children, namely gender and age [13]. Several studies have shown that girls report higher levels of anxiety than boys [6,14]. It has been suggested that assessment of anxiety in youth requires a multi-method, multi-informant approach, drawing information from interviews, youth self-reports, parent and teacher reports, and behavioral observations [12]. An example of this approach is using the School Anxiety Scale-Teacher Report (SAS-TR). It was developed by Lyneham et al. and designed to assess the behavior of 5 to 12 year-old children at school, targeting the behaviors and feelings distinctive to the experience of anxiety. Psychometric evaluation of the original SAS-TR was conducted on 240 community and 140 anxiety disorder children (n = 380). Factor analysis of the SAS-TR identified two subscales reflecting social and generalized anxiety that jointly accounted for 58 % of the variance observed. Also the SAS-TR was found to have an acceptable internal consistency (Crobnbach’s alpha coefficient for the scale was 0.93 and for its subscales social and generalized anxiety it was 0.92 and 0.90, respectively). In order to compare the SAS-TR scores across gender and age groups, the results obtained from univriate analysis of variance indicted no significant evidence for interaction or main effects for age and gender. In addition clinical application of the SAS-TR was evident where the scale discriminated well between the community and clinical groups [15]. Since the SAS-TR was not available in Iran, the present study aimed to translate and culturally adapt the SAS-TR in Iran. The second objective of the study was to investigate the psychometric properties of the Iranian version of SAS-TR.

## Methods

### The questionnaire and scoring

As mentioned earlier the School Anxiety Scale-Teacher Report (SAS-TR) contains two subscales: social anxiety and generalized anxiety. Social anxiety disorder, also known as social phobia (SP), is described as a fear of humiliation and/or embarrassment in social situations, which may lead to significant avoidance of and distress in such situations. Social anxiety in children may be expressed by crying, temper tantrums, fidgeting, somatic complaints, and withdrawal from social situations (e.g., school refusal) [16-18]. Generalized anxiety disorder, formerly termed overanxious disorder, refers to excessive anxiety and worry, accompanied by symptoms of motor tension and vigilance [16,17]. These two subscales include 16 items (7 items on social anxiety and 9 items on generalized anxiety) and it takes approximately 5 minutes to complete the questionnaire. Each item is rated by a teacher to describe how the child had been over the last 6 months on a 4-point Likert scale ranging from 0 to 3 (never = 0, sometimes = 1, often = 2 and always = 3) [15]. Possible scores for the social anxiety range from 0 to 21, for the generalized anxiety from 0 to 27, and for the total anxiety from 0 to 48. Scores of 8 and more on the social anxiety, 10 or more on the generalized anxiety, and 17 or more on the total anxiety is considered to represent high anxious condition.

### Translation

Permission was asked from the main author (Lyneham) to translate and validate the SAS-TR in Iran. Forward-backward procedure was applied to translate the SAS-TR from English into Persian (the Iranian language). Two independent professional translators produced two forward translations of the SAS-TR in Persian. One translator was aware of the project and the other translator was not. Both translators were instructed to aim for conceptual rather than literal translation [19]. Together with one of the authors, the translators compared their translations and produced a single provisional version of the questionnaire. Totally blind to the original version, two other professional translators translated the provisional Persian questionnaire back into the English language [20]. Finally, an expert committee consisting of the translators, the researchers, three psychologists, and one outcome methodologist reviewed the translation and cultural adaptation processes. The authors evaluated all findings from this phase of the adaptation process and then the final Iranian version of SAS-TR was developed and used in this study for further psychometric testing [see Additional file 1.

### Procedure

First a separate list of elementary schools (for girls and boys) were identified in Abhar, Iran. Then using the snowbal approach, four schools for girls and four shcools for boys were selected. After permission from authorities (Misinstry of Education-Abhar Office), the head teachers were contacted in order to coordinate the data collection processes. Prior to the data collection a letter was sent out to all parents explaining the research project and indicating that the during coming days their child might be selected to be evaluated by his or her teacher for indication of any possible anxiety using the attached questionnaire. The Iranian version of SAS-TR was enclosed and they were asked to return the letter by check marking their agreement or disagreement. The head teachers signed the letter and parents were asked to return their reply by a week at latest. The confidentiality clearly was acknowledged in the letter and we indicated that the data would only be used for the research purposes. Then, in an agreed date teachers were asked to complete a paper and pencile version of the final draft of the Iranian version of SAS-TR for each student while one of us (AMo) was present in the agreed school for any possible help or inquiries. Teachers were asked to randomly select five students from the list of students in their class while checking for parental consent. For the purpose of the test-retest analysis four teachers (two male and two female teachers) were asked to complete the questionnaire twice for the same students with a four-week intervals.

### Participants

In all, 200 elementary students aged 6 to 10 years (mean age = 7.97, SD = 1.41) were included in the study. Elementary students divided into two groups: preschool (6 years old, n = 46) and school aged students (7–10 years old, n = 154). Seventy-two percent of elementary students were female (n = 144), and the remaining 28 % were male (n =56). Overall 11 male teachers and 29 female teachers helped us to collect the data. As agreed each teacher was responsible for completing the questionnaire for five students. However, one male teacher completed 6 questionnaires and one female teacher completed 4 questionnaires.

### Statistical analysis

In addition to descriptive statistics several analyses were carried out in order to assess the psychometric properties of the Iranian version of SAS-TR.

*1. Reliability:* Internal consistency of the scale was examined using Cronbach’s alpha coefficient. Values equal to or greater than 0.70 were considered satisfactory [21]. In addition the test-retest reliability of the scale was estimated by intraclass correlation coefficient (ICC). The ICC is an estimate of the fraction of the total measurement variability due to variation among individuals [22]. The following categories were used to interpret the agreement levels: 0.0-0.2 as slight, 0.21-0.40 as fair, 0.41-0.60 as moderate, 0.61-0.80 as substantial, and 0.81-1 as almost perfect [23].

*2. Validity:* It was assessed using content validity. Content validity is the degree to which a sample of items taken together, constitute an adequate operational definition of a construct [24]. For content validity the questionnaire was provided to 10 faculty and psychiatric experts and were asked to answer a 4-point Liker scale in order to assess that the questions measured what they were intended to measure (relevancy), and that the items were clear enough to be understood without difficulty (clarity), and finally that the questions were simple enough to be rated (simplicity). Then, the Content Validity Index (CVI) was calculated for the scale. Polite and Beck recommended 0.80 for the acceptable lower limit for the CVI value [25].

*3. Factor analysis:* The factor structure of the questionnaire was extracted by performing both exploratory (EFA) and confirmatory factor analyses (CFA). Exploratory factor analysis was performed using the principal component analysis with varimax solution. This is a rotation method that minimizes the number of variables that have high loadings on each factor. It simplifies the interpretation of the factors. It was hypothesized that a two-factor solution would be obtained with eigenvalues greater than 1. In order to evaluate sampling adequacy for performing a satisfactory factor analysis, Kaiser-Meyer-Olkin Measure of Sampling Adequacy (KMO) and Bartlett’s tests also were calculated. Confirmatory factor analysis was performed while a two-factor model (generalized anxiety and social anxiety) was specified. Several goodness-of- fit indicators including: goodness of fit index (GFI), adjusted goodness of fit index (AGFI), the root mean square error of approximation (RMSEA), normed fit index (NFI), and comparative fit index (CFI) were selected for reporting the analysis outcomes. The GFI and AGFI are chi-square based calculations independent of degrees of freedom. The recommended cut-off values for acceptable values are ≥ 0.90. The RMSEA tests the fit of the model to the covariance matrix. As a guideline, values of < 0.05 indicate a close fit and values below 0.11 are an acceptable fit. The NFI and CFI values range from 0 to 1 with a value of 0.90 and greater being acceptable fit to the data [26,27].

### Ethics

The study received approval from the Ethics Committee of Bagiyatallah University of Medical Sciences and authorities. In addition, as indicated parental consent was obtained.

## Results

### Age and sex differences

The mean SAS-TR score by age and gender are shown in Table 1. There were significant age differences for the social (P = 0.03) and the total anxiety scores (P = 0.05) but not for the generalized anxiety (P = 0.18). No significant gender differences were observed for the social (P = 0.45) and the total anxiety score (P = 0.74). However, the difference for the generalized anxiety score was just significant (P = 0.05).

**Table 1:** The SAS-TR score by age and gender*

	**Social anxiety**	**Generalized anxiety**	**Total**
	**Mean (SD)**	**Mean (SD)**	**Mean (SD)**
**Age (year)**			
Preschool (6 years old, n = 46)	7.4 (6.2)	6.3 (5.4)	13.7 (11.1)
School age (7–10 years old, n = 154)	5.3 (5.0)	4.7 (4.4)	10.1 (8.9)
P**	*0.03*	*0.18*	*0.05*
**Gender**			
Female (n = 144)	5.9 (5.9)	5.4 (4.8)	11.3 (10.2)
Male (n = 56)	5.5 (3.8)	4.1 (4.1)	9.6 (7.3)
P*	*0.45*	*0.05*	*0.74*

* Higher scores represent greater distress.

** Derived from Mann–Whitney *U* test.

Considering the recommended cut-off values, overall the prevalence of high anxiety condition was found to be 21 %. The results are shown in Table 2. There were significant differences between the two age groups and the different levels of social (P = 0.04), generalized (P =0.01), and total anxiety (P = 0.03). However, there were no significant differences between gender and the different levels of social (P = 0.24), generalized (P = 0.21), and total anxiety (P = 0.14).

**Table 2:** Prevalence of anxiety levels in elementary students according to the cut-off values by age and gender

	**Social anxiety**		**Generalized anxiety**		**Total score**	
	**Normal No. (%)**	**High No. (%)**	**Normal No. (%)**	**High No. (%)**	**Normal No. (%)**	**High No. (%)**
**Age**						
Preschool age (6 years old, n = 46)	30 (19.6)	16 (34.0)	31 (19.4)	15 (37.5)	31 (19.6)	15 (35.7)
School age (7–10 years old, n = 154)	123 (80.4)	31 (66.0)	129 (80.6)	25 (62.5)	127 (80.4)	27 (64.3)
*P (df, χ*^*2*^*)*	*0.04 (1, 4.23)*		*0.01 (1, 5.93)*		*0.03 (1, 4.85)*	
**Gender**						
Female (n = 144)	107 (69.9)	37 (78.7)	112 (70.0)	32 (80.0)	110 (69.6)	34 (81.0)
Male (n = 56)	46 (30.1)	10 (21.3)	48 (30.0)	8 (20.0)	48 (30.4)	8 (19.0)
*P (df, χ*^*2*^*)*	*0.24 (1, 1.37)*		*0.21 (1, 1.58)*		*0.14 (1, 2.11)*	
**All** (n = 200)	153 (76.5)	47 (23.5)	160 (80.0)	40 (20.0)	158 (79.0)	42 (21.0)

### Reliability

The descriptive findings for the social, generalized, and total anxiety scores for all students are presented in Table 3. The internal consistency (to examine reliability) as measured by Cronbach’s alpha coefficient found to be satisfactory. As shown in the Table 3, all alpha coefficients exceeded the recommended value of 0.70, lending support to the scale’s reliability in general and for its subscales in particular. Intraclass correlation coefficients also were found to be acceptable, ranging from 0.70 to 0.92.

**Table 3:** The descriptive findings (n = 200)

	**Mean (SD)**	**Observed range (possible range)**	**Cronbach’s alpha coefficient**	**ICC**
**Social anxiety**	5.1 (4.7)	0-19 (0–21)	0.85	0.92
**Generalized anxiety**	5.8 (5.4)	0-23 (0–27)	0.88	0.70
**The total**	10.9 (9.5)	0-42 (0–48)	0.92	0.81

### Content validity

The content validity index (CVI) for the scale was calculated and it was found to be 0.90 indicating a satisfactory result. Ten experts rated almost all items as relevant, clear and simple.

### Factor analysis

Exploratory factor analysis (EFA) was used to determine the underlying factor structure of the set of items. The calculated KMO was 0.92 and the Bartlett's test of sphericity was significant (P < 0.0001) showing that the sample size was adequate for the analysis. Based on eigenvalues higher than 1 and loading level of 0.4 or above, a two-factor solution emerged. The two-factor solution jointly explained 55.3 % of the total variance observed. The results are shown in Table 4.

**Table 4:** The results obtained from exploratory factor analysis of the Iranian version of SAS-TR

	**Factor1 (Generalized anxiety)**	**Factor2 (Social anxiety)**
1. This child is afraid of asking questions in class.	0.284	**0.647**
2. This child speaks only when someone asks a question them.	0.183	**0.707**
3. This student worries about what others think of him/her.	**0.512**	0.465
4. This child does not volunteer answers or comments during class	0.078	**0.715**
5. This child is afraid of making mistakes.	**0.549**	0.525
6. This child hates being the center of attention.	0.406	**0.620**
7. This child hesitates in starting tasks whether they understood the task before starting.	**0.603**	0.552
8. This child worries about things.	**0.527**	0.485
9. This child worries that (s)he will do badly at school.	**0.601**	0.435
10. This child worries that something bad may happen to him/her.	**0.710**	0.297
11. This child seems very shy.	0.456	**0.553**
12. This child complains of headache, stomach aches or feeling sick.	**0.600**	0.238
13. This child feels afraid when (s)he has to talk in front of the class.	0.499	**0.614**
14. This child hesitates to speak when in group situations.	0.563	**0.617**
15. When this child has a problem, (s)he feels shaky.	**0.764**	0.234
16. This child appears nervous when approached by other children or adults.	**0.667**	0.208
**Eigenvalues**	*7.4*	*1.2*
**Variance observed (%)**	*47.6*	*7.7*

**Figure 1:**
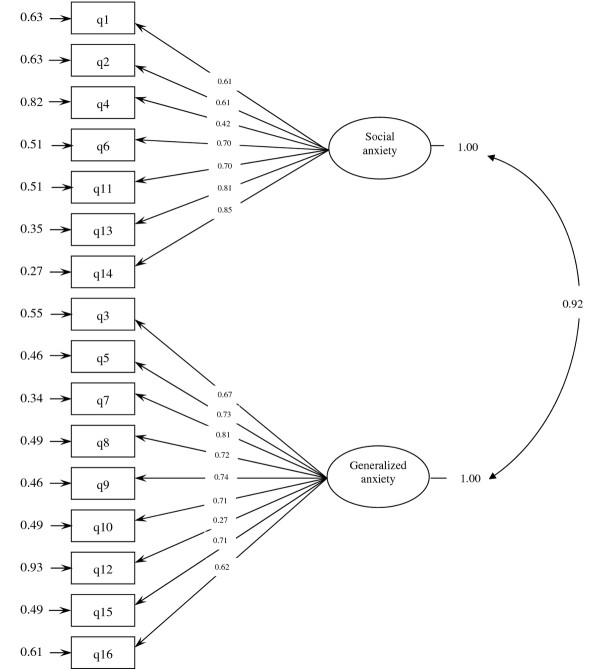
A two-factor model for the Iranian version of SAS-TR obtained from confirmatory factor analysis.

The results for confirmatory factor analysis are shown in Figure 1. The two-factor model, that is generalized and social anxiety, was specified and tested. The results provided a good fit to the data lending support to the original hypothesized structure of the questionnaire with GFI = 0.90, AGFI = 0.82, RMSEA = 0.08, NFI = 0.95, and CFI = 0.96.

## Discussion

The main purpose of this study was to translate and examine reliability and validity of the SAS-TR questionnaire in Iran. In general, the results showed that the Iranian version of SAS-TR had reasonably satisfactory internal consistency, test-retest reliability, content validity and factor structure.

The overall prevalence of total anxiety in elementary students as reported by teachers was found to be 21 %. In fact this indicates that application of the clinical cut-offs designed to identify the top 20 % of anxious children in the Australian data have successfully identified the same proportion of anxious children in the Iranian sample [15]. Also the result was very similar to the findings by other investigators. Egger and Angold [28] found that the prevalence of any psychiatric disorders among 2 to 5 years old preschool children ranged from 16 % to 26 % and Birmaher et al. [29] reported that the prevalence of anxiety in preschool children was 22 %.

The current study did not show significant differences for the total anxiety (P = 0.45) and social anxiety (P = 0.74) among male and female students. These findings were similar to the original validation study that carried out by Lyneham et al. [15], but as indicated by them the findings were different from studies that reported higher levels of anxiety among girls than boys [7,11,13,14]. One explanation for such findings by Lyneham and us might be due to the fact that we both used a proxy measure of anxiety while others used self-rated questionnaires. However, boys scored lower indicating less anxiety than girls and that we found only a marginal significant difference for generalized anxiety between boys and girls (P = 0.05).

The findings also indicated that there were significant differences between age and the total anxiety and social anxiety scores but not for generalized anxiety where preschool children showed higher anxiety. Using the same instrument Lyneham et al. found no significant age differences for the total SAS-TR score or for the scores on the either subscales [15]. To explain our findings as suggested, it is argued when children reach a certain level of cognitive development, their ability to perceive situations as potentially dangerous increases. With increasing age, however, the child gets a better understanding of these situations and learns to control them, which reduces anxiety [13]. In addition, it should be noted that the SAS-TR was designed for school age children and not for preschoolers. Perhaps differences in educational systems may also account for differences in findings. Furthermore, as pointed out in the literature it is generally assumed that anxiety decreases in non-clinical children as they become older [14]. In general there is minimal information about the prevalence of anxiety disorders in preschoolers [30].

Exploratory factor analysis indicated a two-factor solution for the scale that jointly explained 55.3 % of the variance observed. The finding was very similar to the original validation study where a two-factor solution accounting for 58.5 % of the variance was reported (factor 1: 49.6 % and factor 2: 8.9 %) [15]. Interestingly the results obtained from the confirmatory factor analysis also indicated that the two-factor model fitted to the data very well and ensured the original conceptual model of the instrument.

This study had several limitations. The most important one relates to the fact that not many boys included in the study. Secondly we did not use clinical samples or clinical measures to assess further psychometric properties of the scale such as known groups comparison. Thirdly, construct, convergent and divergent validity were not examined. Future studies might focus on these aspects of the validation process.

## Conclusion

This study showed satisfactory cultural adaptation, reliability, content validity and factor structure for the Iranian version of SAS-TR. However, considering the study limitations, the findings should not be generalized. In general this instrument will be a valuable teacher reported measure for the evaluation of school anxiety (social and generalized) among elementary students in Iran and other Persian-speaking countries.

## Competing interests

The authors declare that they have no competing interests.

## Authors’ contribution

AMo was the main investigator and collected the data and wrote the first draft. ZH supervised the study and contributed to the analysis and writing. AE and AFA were the study advisors. MT performed confirmatory factor analysis and provided the figure. AM reanalyzed the data, critically evaluated the manuscript and provided the final manuscript. All authors read and approved the final manuscript.

## Pre-publication history

The pre-publication history for this paper can be accessed here:

http://www.biomedcentral.com/1471-244X/12/82/prepub

## Supplementary Material

Additional file 1 **Iranian (Persian) version of the SAS-TR.** The file contains the Iranian version of the School Anxiety Scale-Teacher Report. (DOC 41 kb) Click here for file

## References

[B1] Cartwright-Hatton S McNicol K Doubleday E Anxiety in a neglected population: prevalence of anxiety disorders in pre-adolescent children Clin Psychol Review 2006 27 817 833 10.1016/j.cpr.2005.12.002 16517038

[B2] Waters AM Craske MG Bergman RL Naliboff BD Negoro H Ornitz EM Developmental changes in startle reactivity in school-age children at risk for and with actual anxiety disorder Int J Psychophysiol 2008 70 158 164 10.1016/j.ijpsycho.2008.07.014 18718853

[B3] Essau CA Frequency, comorbidity and psychosocial impairment of anxiety disorders in German adolescents J Anxiety Disord 2000 14 263 279 10.1016/S0887-6185(99)00039-0 10868984

[B4] Linyan S Kai W Fang F Yi S Xueping G Reliability and validity of the screen for child anxiety related emotional disorders (SCARED) in Chinese children J Anxiety Disord 2008 22 612 621 10.1016/j.janxdis.2007.05.011 17628391

[B5] Spence SH Barrett PM Turner CM Psychometric properties of the Spence Children’s Anxiety Scale with young adolescents Anxiety Disord 2003 17 605 625 10.1016/S0887-6185(02)00236-0 14624814

[B6] Essau CA Sakano Y Ishikawa S Sasagawa S Anxiety symptoms in Japanese and in German children Behav Res Ther 2004 42 601 612 10.1016/S0005-7967(03)00164-5 15033504

[B7] Broeren S Muris P Psychometric evaluation of two new parent-rating scales for measuring anxiety symptoms in young Dutch children J Anxiety Disord 2008 22 949 958 10.1016/j.janxdis.2007.09.008 17977693

[B8] Kessler RC Berglund P Demler O Jin R Merikangas KR Walters EE Lifetime prevalence and age-of-onset distributions of DSM-IV disorders in the National Comorbidity Survey Replication Archives of General Psychiatry 2005 62 593 602 10.1001/archpsyc.62.6.593 15939837

[B9] Whiteside SP Brown AM Exploring the utility of the Spence Children’s Anxiety Scales parent- and child-report forms in a North American sample J Anxiety Disord 2008 22 1440 1446 10.1016/j.janxdis.2008.02.006 18395408

[B10] Muris P Normal and abnormal fear and anxiety in children and adolescents 2007 Elsevier, Oxford

[B11] William Li Ho C Lopez V Development and validation of a short form of the Chinese version of the State Anxiety Scale for Children Int J Nurs Studies 2007 44 566 573 10.1016/j.ijnurstu.2005.12.004 16464452

[B12] Kendall PC Puliafico AC Barmish AJ Choudhury MS Henin A Treadwell KS Assessing anxiety with the Child Behavior Checklist and the Teacher Report Form J Anxiety Disord 2007 21 1004 1015 10.1016/j.janxdis.2006.10.012 17270388

[B13] Bodden D HM, Bogels SM, Muris P: The diagnostic utility of the screen for child anxiety related emotional disorders-71 (SCARED-71) Behav Res Ther 2009 47 418 425 10.1016/j.brat.2009.01.015 19230863

[B14] Castellanos D Hunter T Anxiety disorders in children and adolescents S Med J 1999 92 946 953 10548164

[B15] Lyneham HJ Street AK Abbott MJ Rapee RM Psychometric properties of the school anxiety scale- teacher report (SAS –TR) J Anxiety Disord 2008 22 292 300 10.1016/j.janxdis.2007.02.001 17339095

[B16] Muris P Schmidt H Merckelbach H Correlations among two self-report questionnaires for measuring DSM-defined anxiety disorder symptoms in children: the Screen for Child Anxiety Related Emotional Disorders and the Spence Children's Anxiety Scale Pers Individ Differences 2000 28 333 346 10.1016/S0191-8869(99)00102-6

[B17] Muris P Merckelbach H Ollendick T King N Bogie N Three traditional and three new childhood anxiety questionnaires: their reliability and validity in a normal adolescent sample Behav Res Ther 2002 40 753 772 10.1016/S0005-7967(01)00056-0 12074371

[B18] Kuusikko S Pollock-Wurman R Ebeling H Hurtig T Joskitt L Mattila ML Jussila K Moilanen I Psychometric evaluation of social phobia and anxiety inventory for children (SPAI-C) and social anxiety scale for children-revised (SASC-R) Eur Child Adolesc Psychiatry 2009 18 116 124 10.1007/s00787-008-0712-x 18807111

[B19] WHO Process of translation and adaptation of instruments , [http://www.who.int/substance_abuse/research_tools/translation/en/]

[B20] Bulliger M Alonso J Apolone G Translating health status questionnaire and evaluating their quality: the IQOLA Project approach J Clin Epidemol 1998 9 13 23 10.1016/s0895-4356(98)00082-1 9817108

[B21] Nunnally JC Bernstein IH Psychometric theory 1994 McGraw-Hill Inc, New York

[B22] Anastasia A Validity: Basic Concepts 1990 Macmillan Publishing Company, New York 139 157

[B23] Landis JR Koch GG The measurement of observer agreement for categorical data Biometrics 1977 33 159 174 10.2307/2529310 843571

[B24] Polit DF Beck CT The content validity index: are you sure you know what’s being reported? Critique and recommendations Res in Nurs & Health 2006 29 489 497 10.1002/nur.20147 16977646

[B25] Polit DF Beck CT Nursing research: principles and methods 2004 46 Lippincott, Philadelphia 416 445

[B26] Marsh HW Hau K Wen Z In search of golden rules: comment on hypothesis testing approaches to setting cut-off values for fit indexes and dangers in over generalizing Hu and Bentler’s findings Structural Equation Model 2004 11 320 341 10.1207/s15328007sem1103_2

[B27] Byrne BM Structural Equation Modelling 1998 Lawrence Erlbaum Associates Publishers, Mahwah, NJ

[B28] Egger HL Angold A Common emotional and behavioral disorders in preschool children: Presentation, nosology, and epidemiology J Child Psychol Psychiatry 2006 47 313 337 10.1111/j.1469-7610.2006.01618.x 16492262

[B29] Birmaher B Ehmann M Axelson DA Goldstein BI Monk K Kalas C Kupfer D Kay Gill M Leibenluft E Bridge J Guyer A Egger HL Brent DA Schedule for affective disorders and schizophrenia for school-age children (K-SADS-PL) for the assessment of preschool children: A preliminary psychometric study J Ppsychiatr Res 2009 43 680 686 10.1016/j.jpsychires.2008.10.003 PMC2736874 19000625

[B30] Spence SH Rapee R McDonald C Ingram M The structure of anxiety symptoms among preschoolers Behav Res Ther 2001 39 1293 1316 10.1016/S0005-7967(00)00098-X 11686265

