# Supplementary material for: The School Anxiety Scale-Teacher Report (SAS-TR): translation and psychometric properties of the Iranian version
Source: BMC Psychiatry. 2012 Jul 18;12:82. doi: 10.1186/1471-244X-12-82 (PMC3412721; doi:10.1186/1471-244X-12-82)
Supplement: Additional file 1 — Iranian (Persian) version of the SAS-TR. The file contains the Iranian version of the School Anxiety Scale-Teacher Report. (DOC 41 kb) [file 1471-244X-12-82-S1.doc]

**گونه ايراني مقياس اضطراب مدرسه-گزارش معلم**

**School Anxiety Scale-Teacher Report**

**لطفاً براي هر سؤال بهترين توصيف را درباره چگونگي وضعيت دانش‌آموز در طول 3 ماه گذشته يا در طول اين سال تحصيلي با علامت ضربدر(×) مشخص كنيد. لطفاً به همه سؤالات پاسخ دهيد.**

**هرگز گاهي اوقات اغلب هميشه**

1- اين دانش‌آموز از سؤال كردن در كلاس مي‌ترسد.

2- اين دانش‌آموز تنها زماني صحبت مي‌كند كه كسي از او سؤالي بپرسد.

3- اين دانش‌آموز از اينكه ديگران در مورد او چه فكر مي‌كنند نگران مي‌شود.

4- اين دانش‌آموز در كلاس براي پاسخ به سؤالات يا اظهار نظركردن داوطلب نمي‌شود.

5- اين دانش‌آموز از اشتباه كردن مي‌ترسد.

6- اين دانش‌آموز از اينكه مركز توجه قرار گيرد، متنفر است.

7- اين دانش‌آموز قبل از شروع هر كاري يا براي پرسيدن سؤال دچار ترديد مي‌شود.

8- اين دانش‌آموز درباره موضوعات مختلف نگران است.

9- اين دانش‌آموز نگران اين است كه كارهاي مدرسه‌اش را بد انجام دهد.

10- اين دانش‌آموز نگران است كه اتفاق بدي براي او رخ دهد.

11- اين دانش‌آموز خيلي خجالتي به نظر مي‌آيد.

12- اين دانش‌آموز اظهار سردرد، درد معده و احساس بيماري مي‌كند.

13- اين دانش‌آموز وقتي كه بايد در كلاس صحبت كند احساس ترس مي‌كند.

14- اين دانش‌آموز، در موقعيت‌هاي گروهي از صحبت كردن اجتناب مي‌كند.

15- وقتي اين دانش‌آموز مشكلي داشته باشد دچار ترس و لرز مي‌شود.

16- اين دانش‌آموز هنگامي كه ساير كودكان يا بزرگسالان به او نزديك مي‌شوند عصبي به نظر مي‌رسد.

© Center for Emotional Health, Macquarie University, Sydney

www.ceh.mq.edu.au

پژوهشكده علوم بهداشتي جهاد دانشگاهي
